# Individualizing pediatric boarding care: child perspectives

**DOI:** 10.1186/s12887-025-05748-9

**Published:** 2025-07-02

**Authors:** Emily D. Irwin, Briana P. Keller, Andrea Hughie, Patti Runyan, Jessika C. Boles

**Affiliations:** 1 Vanderbilt University, Nashville, TN USA; 2 Monroe Carell Jr. Children’s Hospital at Vanderbilt, Nashville, TN USA

**Keywords:** Psychiatric boarding, Behavioral health, Pediatric individualized care planning, Mental health

## Abstract

**Background:**

Hundreds of thousands of pediatric patients receive inpatient behavioral health care each year, and many of these experience boarding stays in general inpatient units while they await transfer to a psychiatric facility. Boarding stays are often characterized by low quality of care, including a lack of individualized care planning. The authors present an evaluation of an individualized care planning tool, the My Health Passport, reported by pediatric patients to understand the perceptions and feasibility of implementation.

**Methods:**

A total of 100 pediatric patients ages 8–17 who were admitted for behavioral health concerns and experienced boarding participated. Following completion of the My Health Passport tool, participants completed a questionnaire regarding their helpfulness, satisfaction, and suggestions for improving the tool. A prospective cohort design was used. Quantitative data were analyzed via descriptive statistics utilizing SPSS, and an inductive coding process was used to evaluate short-answer responses.

**Results:**

Data suggest pediatric patients were largely satisfied with the tool, with a mean satisfaction rating of 7.72 out of 10. Patients find it improves their stay through increasing comfort with staff and emphasizing the use of coping skills with tailored questions. The possibility of creating multiple forms of the tool, with varying levels of complexity, was raised. Participants also envisioned My Health Passport to be a useful tool in settings such as their homes, schools, community organizations, and other facilities in which they may receive inpatient psychiatric care.

**Conclusion:**

Overall, this tool is received well and perceived as helpful by pediatric patients. Future research could evaluate the use of an individualized behavioral health planning tool in outpatient settings and with longitudinal cohorts.

## Background

As reported by the American Academy of Pediatrics, due to exceptionally long wait times for outpatient behavioral health care, about 500,000 children with mental or behavioral health disorders are seen in emergency departments each year for reasons including psychosis, aggression, self-harm, suicide attempts, substance use disorders, and other conditions [1]. Since the beginning of the COVID-19 pandemic in 2020, the number of pediatric patients seeking behavioral health treatment has substantially increased [2, 3]. Due to limited capacity, many children and their families face extended stays in the emergency department or general inpatient pediatric units as they await placement in an appropriately resourced psychiatric facility, a practice known as boarding [4]. These stays can be described as unstructured and not therapeutic, lasting anywhere from hours to several days [4–6]. Younger patients are at particular risk of facing extended boarding stays due to the limited quantity of available services for this age group [5].

Boarding stays typically do not offer adequate psychological or developmental support for this patient population, as demonstrated by low levels of reported quality of care [2, 6]. One especially prevalent challenge is the difficulty of individualized care planning. While personalized care planning has been well-established and studied in pediatrics for vaccine or needle procedures, pain reduction, and treatment of patients with developmental disabilities [7–9], its application has not previously been explored in the pediatric behavioral health population. This lack of individualized care is often a reported point of frustration among both patients and their caregivers [10, 11].

Previous research has shown that children with behavioral health needs, like other pediatric healthcare populations, desire involvement in their care planning and decision-making processes [10, 12]. In fact, when children with mental and behavioral health needs are involved in their care, they report more favorable experiences [12]. Additionally, higher levels of satisfaction with inpatient psychiatric care are closely related to children’s levels of engagement in treatment after hospital discharge, suggesting a decreased risk of emergency room presentation in the future [13]. In addition to increasing patient satisfaction, patient engagement and care planning supports are also intended to assist emergency room staff, who serve as the “front line” of defense for acute behavioral health patients; yet these healthcare professionals frequently report feeling under-prepared to deliver these types of supports [2, 14]. While many children and adolescents may struggle to verbally communicate their needs and concerns to healthcare providers, they may find it easier to do so using alternative mediums such as written communication [15].

Though pediatric behavioral health boarding stays may last only a few days or less [4], they typically occur when a child’s psychological distress is most intense, thereby making this a significantly vulnerable period for the child’s developmental and psychosocial well-being. As emotions are high, behaviors are likewise less predictable, more intense, and can frequently incur patient or staff injuries [16]. As patient and staff safety continue to rise to the forefront of healthcare quality metrics and workplace well-being indices [17], quick to implement short-term modifications like the My Health Passport program may be particularly useful in this context.

The increase in children’s behavioral health needs and lack of resources demonstrate a necessity to identify patient- and family-centered interventions that help support this population during boarding hospital stays. Although personalized care and child engagement appear to be protective factors in this context [10, 12], little has been done to translate these into interventions that can be studied. Therefore, the purpose of this study was to examine pediatric patients’ experiences and perceptions with an individualized care planning tool aimed at supporting boarding stays.

## Methods

A prospective cohort design was used to optimize the quality of data that could be gathered from pediatric participants actively hospitalized for an acute behavioral health concern. With a retrospective design, there would be a risk of perception loss due to the administration of treatment and passage of time. Additionally, as the My Health Passport is already clinically implemented at the research site for its utility during the child’s hospitalization, it was most efficient and least disruptive to align data collection procedures with the tool’s completion and implementation in the child’s clinical care.

### Site

The research site was a freestanding children’s hospital associated with an academic medical center in the southeastern United States. It is a designated Level I trauma center housing more than 400 inpatient beds, which includes three intensive care units. There is no dedicated psychiatric floor or service at this institution; instead, pediatric patients are transferred to one of four psychiatric facilities in the metropolitan area. These facilities are typically at capacity, thus patients in need of inpatient psychiatric care may board at the research site for anywhere from 24 h to several days, depending on placement availability. While patients await transfer, they are under the care of a primary psychologist who manages safety planning and disposition planning (coordinating acceptance and transfer to specific behavioral facilities when indicated) and conducts assessments that include feedback from the patient when possible and parents/caregivers. Patients also receive a one-to-one mental health specialist for continuous monitoring when the patient appears to be a threat to themselves or others. The mental health specialist coordinates the provision of vitals and daily care while ensuring safety and engaging the patient in normalizing activities as much as possible.

Patients are also eligible for a variety of psychosocial services via consultation, such as child life, facility dog, art and music therapies, and spiritual care.

No psychiatric care is initiated until the patient is transferred unless necessary for immediate safety concerns.

### My health passport

**Fig. 1:**
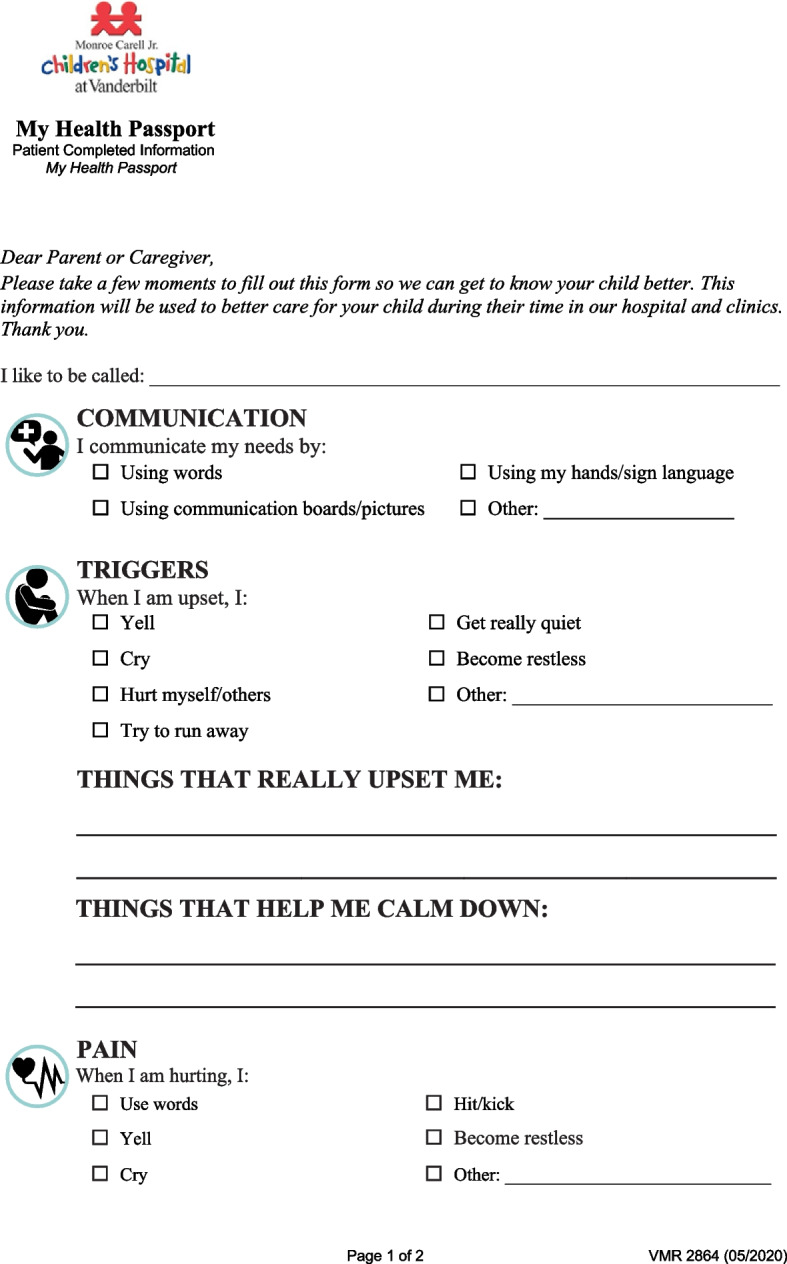
Front side of the My Health Passport Form

**Fig. 2:**
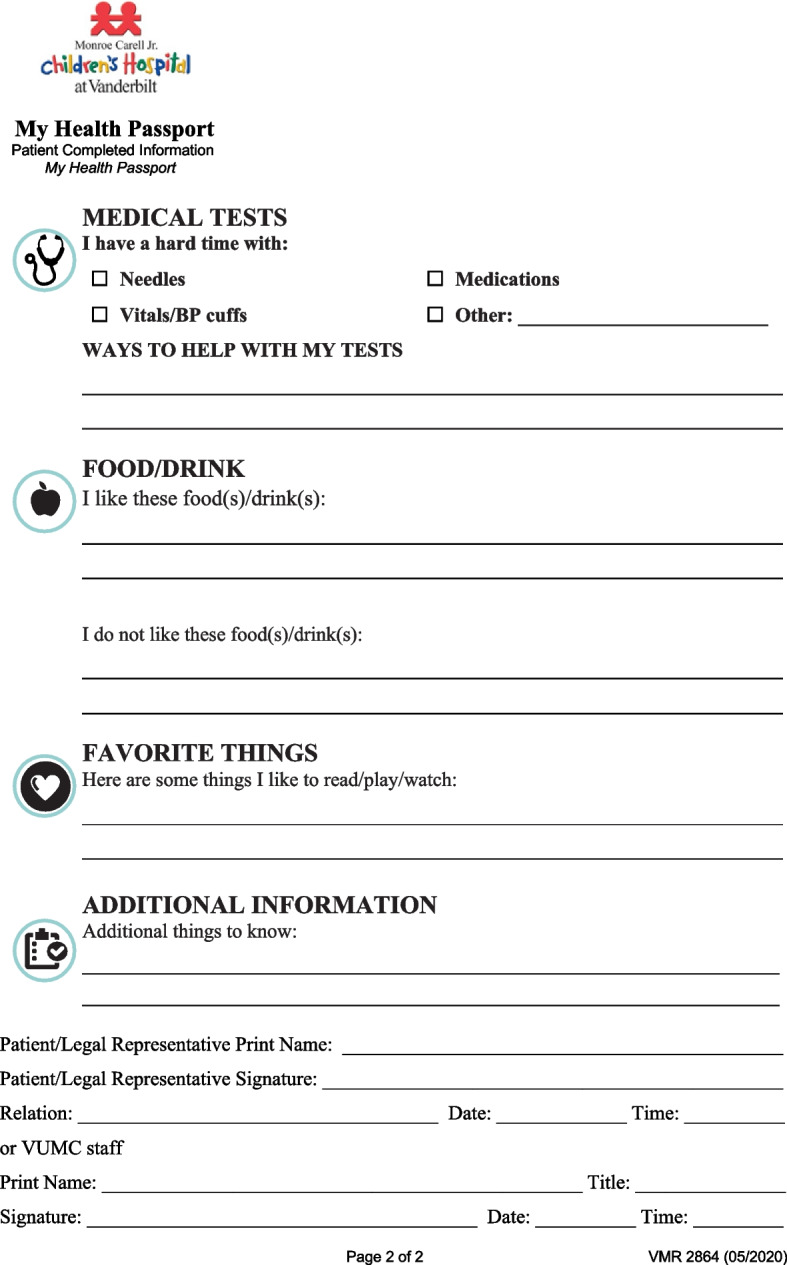
Back Side of the My Health Passport Form

The My Health Passport (MHP) is a behavioral health care planning tool consisting of one double-sided page (see Figs. 1 and 2) and is designed to be filled out by the caregiver or patient at the time they present to the emergency department. The program was designed in 2017 by the multidisciplinary Pediatric Behavioral Health and Intervention Team, which included a nursing director, psychologist, social worker, child life specialist, creative arts therapists, and staff nurses. Development of MHP was drawn from insights gathered in previous research, including care planning tools in other pediatric populations [7–10, 12]. Patients are encouraged to fill out the form after arrival at the hospital to support a more individualized approach to their care while specialized services are limited. Each completed MHP is posted at the bedside and scanned into their electronic health record (EHR) by a member of staff. At this time, the tool demonstrates an 80% completion rate as per an internal quality metric calculated through a retrospective electronic chart auditing procedure to identify which eligible records contain documentation of form completion or an uploaded scan of the completed form.

### Participants

A total of 100 pediatric patients between the ages of 8 and 17 years took part in this study (see Table 1 below) via a convenience sample. All were experiencing inpatient boarding care while awaiting transfer to an inpatient psychiatric facility for behavioral health concerns and had completed a My Health Passport form. All had spent a minimum of 12 h on the inpatient unit at the time of study participation and had been deemed “safe” by the treating physician."Safe” was defined as the patient having experienced at least 24 consecutive hours without causing or attempting to cause harm to themselves or others. For patients who had not been admitted for 24 h, this became up to the discretion of the physician. Safe participants between 8–17 admitted for a behavioral health concern who were fluent in English were included. Participants were only excluded if: 1) they were under state custody, 2) did not speak English as their primary language, or 3) were cognitively unable to participate due to developmental or illness reasons. Of the 100 who participated, 70% reported having a previous hospital stay at the research site, with 52% reporting a prior inpatient stay at another facility.

**Table 1:** Participant demographics

Variables	(n)	Percentage
**Age**		
8 to 10	9	9.0
11 to 12	14	14.0
13 to 15	41	41.0
16 to 18	36	36.0
**Gender**		
Male	33	33.3
Female	48	48.5
Nonbinary	9	9.1
Transgender	4	4.0
Other	5	5.1
**Race**		
White/Caucasian	54	57.4
Black/African American	26	27.7
American Indian/Native American	1	1.1
Asian American/Pacific Islander	1	1.1
Other	12	12.8

*N* = 100. Not all participants answered all demographic questions

### Procedures

During daily rounds, a member of the site’s multidisciplinary behavioral health team identified inpatients meeting inclusion criteria. A study team member, either a graduate research assistant or behavioral health team member, then sought parent/guardian informed consent before approaching the patient for assent. Most participants completed the assent form and survey independently on REDCap (Research Electronic Data Capture), a secure web application used for building and managing surveys and databases, using an iPad. In cases where this was not feasible (such as an intellectual or developmental disability, or history of aggressive behavior), a research assistant or parent/guardian read the questions aloud and entered the participant’s dictated responses, or participants completed a printed version of the survey, which was later entered into REDCap.

After providing assent, participants completed a brief demographics survey (see Table 1), followed by a short series of questions regarding the MHP tool (see Table 2). These included nine questions that utilized either a five-point Likert scale (where 1 = “Disagree a Lot” and 5 = “Agree a Lot”) or a rating scale of one to 10, and five open-ended written responses (see Table 3). All questions were created by the research team. Data were collected between May 2022 and July 2023.

**Table 2:** Survey Questions

Survey Question				
*The My Health Passport tool…*	***n***	**Range**	***M***	***SD***
Helped my doctors and nurses take good care of me	100	3–5	4.42	.741
Helped me feel safe in the hospital	99	2–5	4.39	.831
Was easy for me to understand	99	1–5	4.38	.779
Helped my doctors and nurses know what upsets me	99	1–5	4.28	.869
Helped my doctors and nurses know what helps me calm down	99	1–5	4.20	.857
Helped my doctors and nurses know the important things about me	100	3–5	4.18	.796
Helped my doctors and nurses know how to talk to me	99	2–5	4.15	.885
Helped me manage my behaviors in the hospital	99	2–5	4.05	.930
Made my stay at the hospital better	99	1–5	4.04	1.039

**Table 3:** Open-ended response questions

Question
When should doctors and nurses tell kids and families about the My Health Passport form?
How should doctors and nurses tell you about the My Health Passport form?
Where else could you see the My Health Passport form being helpful for you, your family, or your doctors and nurses?
What could we do to make the My Health Passport form better?
Is there anything else you want to tell us about the My Health Passport form?

### Analysis

Descriptive statistics were conducted using IBM SPSS Statistics Version 29. Qualitative data for the open-ended questions were analyzed using a line-by-line, inductive coding approach as per Boles and colleagues (2017) [18]. Coding was conducted individually by two graduate-level research assistants, categorizing statements according to the essence of the response. Themes were then generated based on consensus on the similarity of essence or identification of keywords such as “school” or “helpful.”

## Results

### Quantitative

When asked to rate the overall helpfulness of the MHP on a scale of one (“not very helpful”) to 10 (“very helpful”), a total of 86 participants responded. The mean rating was 7.72 (*SD* = 1.84). Additionally, all Survey items regarding satisfaction with the MHP received mean ratings ranging from 4.04 to 4.42 (see Table 2 below). These scores suggest pediatric patients with behavioral health needs perceive a wide range of benefits associated with the MHP tool.

### Qualitative

Participant responses to open-ended questions centered around four themes: 1) encountering the MHP tool, 2) applications of MHP, 3) perceived benefits of MHP, and 4) recommended adaptations.

#### Perceptions on encountering the MHP tool

Most participants reported they would like to receive information about the MHP as soon as possible after presenting to the hospital, ideally on the day of arrival or the next day, and preferably in the emergency department as the patient’s first point of contact with the healthcare team. Two participants stated the importance of completing the MHP tool prior to any medical procedures involving needles or anticipated discomfort. More specifically, a 16-year-old female participant proposed introducing the MHP “when it is evident that the stay will be long enough for an emotional relationship between patient [and] staff to be established.”

Some participants also emphasized a desire for staff to verbally explain the purpose of the MHP, rather than expecting the form or its role to be self-explanatory. One 13-year-old female participant stated, “nurses and doctors should help you understand the form and help the families know what the form is for.” A 14-year-old, gender non-conforming participant added, “when doctors and nurses come to the room to talk to parents about what is going on, start to explain [the MHP] and introduce it. Explain to the patient, and if they need more help understanding, then explain to the parent too.” Several participants recommended the MHP be described as a “tool” to help staff better understand them and care for them while in the hospital.

#### Perceptions on applications of MHP

Participants described other places where they felt the MHP tool could be useful. Most commonly, they discussed applications to other healthcare facilities such as psychiatric hospitals (*n* = 18), at home with their family (*n* = 9), at school (*n* = 6), or in therapy settings (*n* = 3). One 12-year-old female wanted to utilize it “when moving somewhere new so that people can know more about you,” and another participant thought it would be helpful in a juvenile detention setting.

#### Perceived benefits of MHP

The majority of written responses outlined positive feelings about the MHP. One 13-year-old female participant stated, “The My Health Passport form can help patients feel comfortable with the nurses and doctors when the patient is upset,” thereby recognizing part of the clinical intent of its implementation. A 15-year-old participant who self-identified as non-binary said, “it’s an easy way for the doctors and nurses to learn more about the patients without having to re-explain everything about themselves to everyone they meet…It helped me identify triggers that I have and it helped me think deeply about what I am comfortable and not comfortable with.” Other participants also mentioned this perceived self-reflective or coping value of the MHP tool.

#### Recommended adaptations

A few patients suggested improvements to the MHP. One 16-year-old participant proposed, “possibly expanding on questions more for people who internalize more specific instructions [such] as instead of ‘When I am upset, I…’ The question could be ‘When I am feeling upset, I react by…’ And the answers were replaced with active verbs.” Possible additions ranged from questions about “gender expression” (13-year-old, nonbinary participant) to a section about “supporting people or favorite people” (11-year-old male). Several other participants also discussed the possibility of expanding the MHP tool, such as by “making it more in-depth” (15-year-old male) or adding “more questions for the older group of kids/teenagers” (14-year-old female).

On the converse, some participants critiqued the length of the MHP tool, with one 14-year-old gender non-conforming participant sharing, “it felt complicated.” A 15-year-old, non-binary participant suggested, “maybe adding more color to the worksheet.” Even younger participants had recommendations to share, with one 8-year-old suggesting the MHP tool could ask, “what do you li[k]e to play with,” or include “patient things on the front and things for doctors on the back” to make it, “more simple.”

## Discussion

Previously, efforts to support pediatric behavioral health patients while boarding and awaiting specialized care have lacked personalized and dedicated psychosocial support [10, 14]. Responses from pediatric patients indicate the MHP shows promise as a tool for individualizing their care. Most notably, they perceive that it improves the quality of care they receive from doctors and nurses while also helping them feel safe in the hospital. As satisfaction with the inpatient quality of stay is closely related to pediatric patient engagement in post-discharge treatment, it is suggested that tools that individualize care, such as MHP, could improve outcomes not only during the initial inpatient stay but in the long-term treatment trajectory as well [13], though future research should attempt to identify relationship.

Participants voiced a desire to hear about the MHP as soon as possible after their admission, and to receive a verbal explanation of its purpose. These themes indicate youth see a benefit to having completed the MHP early on. Some responses underlined the need for having MHP completed in advance of any medical tests involving needles, suggesting these tests are easier or less distressing when individualized care has been provided. The benefits of personalized care planning in needlestick procedures have been previously documented, though primarily during vaccination or inpatient admission for chronic health conditions [7, 9]. The results of this study suggest a similar need among pediatric patients with acute or chronic mental health conditions.

Generally, participants indicated that the MHP improved their stay in the hospital, and many felt it would also improve their stay at a psychiatric facility. The second and third most common suggestions for other settings where the MHP would be useful were school and home. These results suggest participants do not feel stigma around the idea of using the MHP in home or community settings, but rather perceive it to be valuable in these contexts. This finding aligns with previous research that has found adolescents may be better able to communicate health-related information when utilizing written communication rather than oral communication [15]. These benefits may exist even outside of the healthcare setting, as prompted by participants’ responses about using MHP in a school, home, or juvenile detention facility.

When asked how they would improve the MHP, participants provided conflicting responses. Some requested a simpler and more colorful form, including questions about favorite people or favorite toys, while others felt the form was too brief and aimed at younger children. These results raise the possibility of offering multiple forms of the MHP or another individualized planning tool, where patients are given the option of completing a simpler form or an expanded form. This further highlights the need for individualized boarding care, as perceptions between participants and patients could be very different when given the same form.

### Limitations

Our demographics skewed slightly towards older participants. The majority of participants in this study were at least 13 years old (77%) and in ninth grade or higher (61%). However, this range does align with the current characteristics of patients who are in boarding for mental health needs. The use of a convenience sample from a single site may also limit variability in results and demographics, including cultural differences such as perception of mental health and psychosocial supports. Sampling bias may also be considered a limitation due to the methodology. Additionally, information on participants’ diagnoses (including type and prevalence of intellectual or developmental disabilities) that did not prohibit participation was intentionally not collected, to humanize the research participant experience rather than enforcing stigma around behavioral health diagnoses or lack thereof. Without this information, the authors recognize this may impact the generalizability of the findings and identification of which patients may most benefit from MHP. Length of stay and time of administering the MHP were also not collected, which are both variables that may impact patient experience. Lastly, the inclusion and exclusion criteria of clinical severity can be interpreted as subjective, suggesting this population may be difficult to precisely replicate.

### Future research

Future research should build upon these findings through future adaptations of the tool, enhanced methodology, and assessment of long-term outcomes. This may be done by utilizing longitudinal cohorts, incorporating developmentally appropriate language and formatting that is more specific to various age groups, and implementation in community-based settings.

## Conclusion

Youth responses to the My Health Passport tool were overall positive and highlight hospitalized children’s desire for individualized care while boarding and awaiting inpatient behavioral health placement. These tools appear to increase satisfaction with the quality of care, which can support engagement in treatment post-hospitalization [14]. Increased collaboration within multidisciplinary teams caring for pediatric behavioral health patients may support these efforts by utilizing each discipline’s unique skill set.

## Data Availability

All data generated or analyzed during this study are included in this published article.

## Abbreviations

MHP: My Health Passport
EHR: Electronic Health Record
REDCap: Research Electronic Data Capture
